# Predicting recurrent chat contact in a psychological intervention for the youth using natural language processing

**DOI:** 10.1038/s41746-024-01121-9

**Published:** 2024-05-18

**Authors:** Silvan Hornstein, Jonas Scharfenberger, Ulrike Lueken, Richard Wundrack, Kevin Hilbert

**Affiliations:** 1 Department of Psychology, Humboldt-Universität zu Berlin, 10099 Berlin, Germany; 2 Institute of Information Systems, Leuphana University, Lueneburg, Germany; 3 German Center for Mental Health (DZPG), partner site Berlin/Potsdam, Potsdam, Germany

**Keywords:** Health care, Public health

## Abstract

Chat-based counseling hotlines emerged as a promising low-threshold intervention for youth mental health. However, despite the resulting availability of large text corpora, little work has investigated Natural Language Processing (NLP) applications within this setting. Therefore, this preregistered approach (OSF: XA4PN) utilizes a sample of approximately 19,000 children and young adults that received a chat consultation from a 24/7 crisis service in Germany. Around 800,000 messages were used to predict whether chatters would contact the service again, as this would allow the provision of or redirection to additional treatment. We trained an XGBoost Classifier on the words of the anonymized conversations, using repeated cross-validation and bayesian optimization for hyperparameter search. The best model was able to achieve an AUROC score of 0.68 (*p* < 0.01) on the previously unseen 3942 newest consultations. A shapely-based explainability approach revealed that words indicating younger age or female gender and terms related to self-harm and suicidal thoughts were associated with a higher chance of recontacting. We conclude that NLP-based predictions of recurrent contact are a promising path toward personalized care at chat hotlines.

## Introduction

Mental disorders are highly prevalent^1^. Being responsible for around a third of all years lived in disability^2^ makes them a major public health concern. While occurring across the lifespan, early life phases appear to be particularly critical for mental health, as a majority of disorders develop within the first 24 years of life^3^, and symptoms are likely to sustain into later life phases^4^. However, most affected young people are not accessing care^5,6^, commonly due to a lack of knowledge, perceived stigma and structural barriers^7^. This makes low-threshold forms of mental health support for the youth highly needed.

One such approach is the provision of counseling through chat-based hotlines^8,9^. First, studies have shown that chat-based synchronous interventions can be an effective treatment and prevention method for children and young adults with diverse mental health symptoms^10–12^. Additionally, those hotlines proved to be a first contact point for the youth^13^, serving as access points into further mental health treatment^14^. Therefore, besides the provision of efficient counseling during the chat interaction, the identification of those in need of additional help and their redirection towards appropriate care is a key task of those hotlines. This makes them a potentially highly relevant actor within a stepped-care approach towards mental healthcare^15^ where treatment is adapted towards the needs of the patients.

Natural Language Processing (NLP), the sub-branch of Artificial Intelligence (AI) dealing with language, seems a highly promising approach for improving those key functionality of chat-based counseling. Language is a rich source of information and machines can potentially extract meaningful information from text above human capabilities^16,17^.

This informational gain could accelerate treatment decisions as shown extensively in the mental health domain. For example, discharge notes from an inpatient unit have been used to predict readmission for patients diagnosed with major depressive disorder^18^. Similarly, clinical notes have been used for detection^19^ and prediction^20^ of suicidal behaviour, the identification of symptom information^21^ and patient stratification^22^. Another popular approach is the utilization of non-clinical data such as social media posts for the detection and monitoring of mental health problems^23^. Finally, NLP approaches have been applied to therapist-patient interactions^24^, such as for the emotional evaluation or the classification of the therapeutic alliance^25^.

Chat counseling services accumulate large bodies of chatdata by providing their services. Therefore, it is surprising that comparably little work has investigated NLP applications within this setting. A study from 2016 applied NLP techniques to identify counseling strategies associated with better outcomes, utilizing a sample of 80.000 text-based counseling conversations^26^. Also, around 500 chat transcripts from an online crisis service for the development of an algorithmic approach for classifying and triaging those chats by their level of distress^27^. Here, the model reached high performances of AUC scores of close to 0.9, however the used test set did just include 78 chats. Another approach used around 5500 chat consultations to detect disclosure of suicidal ideation, using a deep-learning-based model incorporating external domain knowledge^28^. Other work used NLP techniques for the development of chatbots^29–31^.

While the summarized approaches revealed promising results, much more work appears needed toward the implementation of NLP techniques into real-world chat-based counseling setups. In particular, we could not find any work using NLP to support the role of chat-based counseling as a first access point into mental health care. Here, recurrent chat contact has been described as a major pain point of the service providers. Chat capabilities are limited, and heavy users have been shown to block a significant share of counselors’ time^32^. This threatens the ability of the services to provide counseling to all incoming help seekers, leaving those contacting possibly without any support. Also, it remains questionable whether recurrent chat consultations would be the most adequate support for those in need of considerable additional help. Frequent chatters have been shown to have significantly more severe problems^32^. Similar insights have been reported for other low-threshold interventions like telephone helplines^33^. Commonly, psychotherapy, pharmacological treatment and family interventions are named as the main treatment approaches for those struggling with severe psychiatric symptoms^34^. However, chat services can not provide any medication and the possibilities to integrate the social environment of the chatters are highly limited. Also, therapeutic alliance has been shown to be central for outcomes in mental healthcare services for the youth^35^. In contrast, the constant ability of the same counselor for chatters seems very difficult to provide at those 24/7 available and commonly volunteer-based services. Therefore, the evaluation of whether additional support or help is needed after a first interaction seems crucial for the operational abilities of the service, but also for the adequate and timely provision of care to the youth. Those in high chance of recontacting the service could be provided with an automated redirection to routine mental healthcare, saving the service provider capacities and reducing the time to adequate care for the chatter. Alternatively, specialized interventions from the side of the service have been proposed for frequent contacts, including the development of a comprehensive health plan and the assignment to higher intensity treatment options^33^. Such data-based “stepping up” of the level of care has been shown to be a heavily used mechanism for personalized treatment in other settings^36^ with promising results^37^. In conclusion, the application of NLP techniques to predict the unmet need for further help after a first consultation could be a starting point for personalized, stepped treatment to reduce costs and improve outcomes.

Addressing this gap, this paper intends to add to the development of AI applications in chat-based counseling services by evaluating the feasibility of an NLP approach focussing on their role as the first point of contact. Specifically, we use the data of around 19.000 individuals that contacted a 24/7 chat counseling service for children and young adults in Germany. Based on that data, we predict whether those chatters recontacted the service after a first counseling session, as an objectively measurable behavioral marker of unmet need for additional help. We hypothesize that we can reach significant predictive performance on a previously unseen test set of conversations, which would provide a decision base for an informed redirection into additional care.

## Results

### Study population and outcome

Our sample for the prediction of recurrent chat contact consisted of routine-care data from the German 24/7 chat-counseling service “krisenchat”, provided free of charge for children and young adults^13^. The anonymized conversations of all individuals that contacted the organization and received a first consultation between October 2021 and December 2022 were included, resulting in a final sample of 18,871 unique chatters. For those chatters, the whole first consultations were included, therefore the chatters messages as well as those by the counselor. Overall, this sample consisted of around 813,000 messages (333,454 by counselors and 479,782 by chatters) and approximately 8.6 million words. For this sample, 8141 of the 18,871 chatters contacted the service again within 6 months, which was defined as the outcome for the prediction task (see Table 1).

**Table 1:** Sample details

	All Consultations (*N* = 18,871)		Train Data (*N* = 14,929)		Test Data (*N* = 3942)	
	Mean	SD	Mean	SD	Mean	SD
Chatter Messages	25.4	20.8	25.3	21.0	26.0	20.2
Counselor Messages	17.7	21.1	17.6	12.1	18.1	12.3
Word Count	456.4	283.9	449.1	281.3	483.7	292.1
Recurrent Contact (%)	43.1%		43.3%		42.3%	

### Algorithm training

**Fig. 1:**
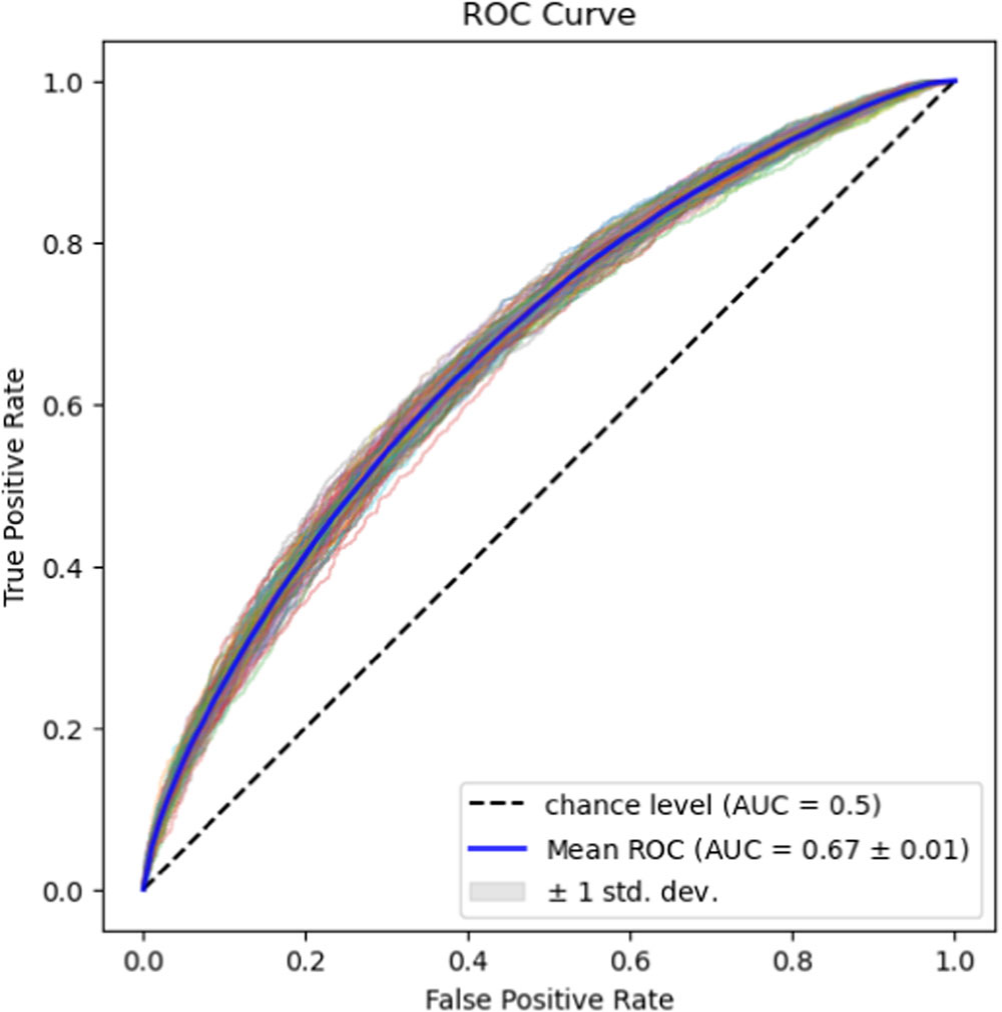
ROC curve of the repeated 5-fold cross-validation for the best hyperparameters. The blue line is showing the averaged performance across all runs, while the grey area represents the standard deviation around this average. The dashed line represents the performance at the chance level.

We divided our sample into a train set of 14,929 first consultations and a test set of 3942 for a final evaluation, applying a time-based split criterion. The train set was used for all decisions regarding hyperparameters and algorithmic specifications. We applied a 5 times repeated 5-fold Cross-Validation and Bayesian hyperparameter search and used, due to the data privacy restrictions, a vectorized word approach for the feature generation out of the strictly anonymized and randomly ordered words of the consultations (see Methods). The best performing set of hyperparameters (max_df_chatter=0.8, min_df_chatter=150, max_df_couns=0.3, mind_df_couns=75, colsample_bytree=0.9, eta= 0.05, gamma=1.5, max_depth=8, min_child_weight=20, subsample = 0.6, no idf, see Table 2 for details on the parameters.) reached an AUROC score of 0.67 over cross-validation (see Fig. 1). Therefore, words that were used by less than 150 chatters were not used in the final model.

**Table 2:** Overview of tuned hyperparameters

	Brief description	Value range	Selected parameter
max_df_chatter	Terms that appear in more chatter documents than the threshold value are ignored. The value represents the proportion of documents.	[0.2, 0.3, 0.4, 0.5, 0.6, 0.7, 0.8, 0.9, 1.0]	0.8
min_df_chatter	Terms that appear in fewer chatter documents than the threshold value are ignored.	[1, 2, 5, 10, 25, 50, 75, 100, 150, 200]	150
max_df_couns	Analogous to max_df_chatter for counselor messages	[0.2, 0.3, 0.4, 0.5, 0.6, 0.7, 0.8, 0.9, 1.0]	0.3
min_df_couns	Analogous to min_df_chatter for counselor messages	[1, 2, 5, 10, 25, 50, 75, 100, 150, 200]	75
colsample_bytree	Subsample ratio of columns for growing trees	[0.2, 0.4, 0.6, 0.7, 0.8, 0.9, 1.0]	0.9
eta	Learning rate	[0.005, 0.01, 0.05, 0.1, 0.2]	0.05
gamma	Minimum loss reduction to make a further split on a leaf node	[0, 0.25, 0.5, 1, 1.5, 2, 5, 10]	1.5
max_depth	Maximum depth of a tree	[2, 4, 6, 8, 10, 12, 14, 16]	8
min_child_weight	Minimum sum of instance weight (hessian) needed in a child	[1, 5, 10, 20]	20
subsample	Subsample ratio of the training instances prior to growing trees	[0.2, 0.4, 0.6, 0.7, 0.8, 0.9, 1.0]	0.6
use_idf	Whether to term frequencies should be reweighted by the inverse document frequencies.	[True, False]	False

In contrast, for the counselor messages frequently used words were excluded, with 30% as maximum occurrence included. While this was better than the approaches for chatter/counselor messages alone, those ones also reached a notable performance of 0.65 each for their best set of hyperparameters. The combined use of chatters and counselors messages without information on who used what words reached an AUROC of 0.66.

As a baseline algorithm, we used the count of the word stems, separated by chatters and counselors, as well as the anonymized time of first contact. We then applied the same procedure outlined above. Here, the best-performing set of hyperparameters (colsample_bytree=0.8, eta= 0.01, gamma=0.25, max_depth=6, min_child_weight=10, subsample = 0.8, see Table 2 for details on the parameters.) achieved an AUROC score of 0.57. Therefore, conversation length as well as daytime alone included some predictive signal regarding the recurrence of contact. A 5 × 2 paired t-test (see Methods) suggested a significant higher performance of the text-data in comparison with this baseline on the train set (*p* < 0.01).

### Independent evaluation

**Fig. 2:**
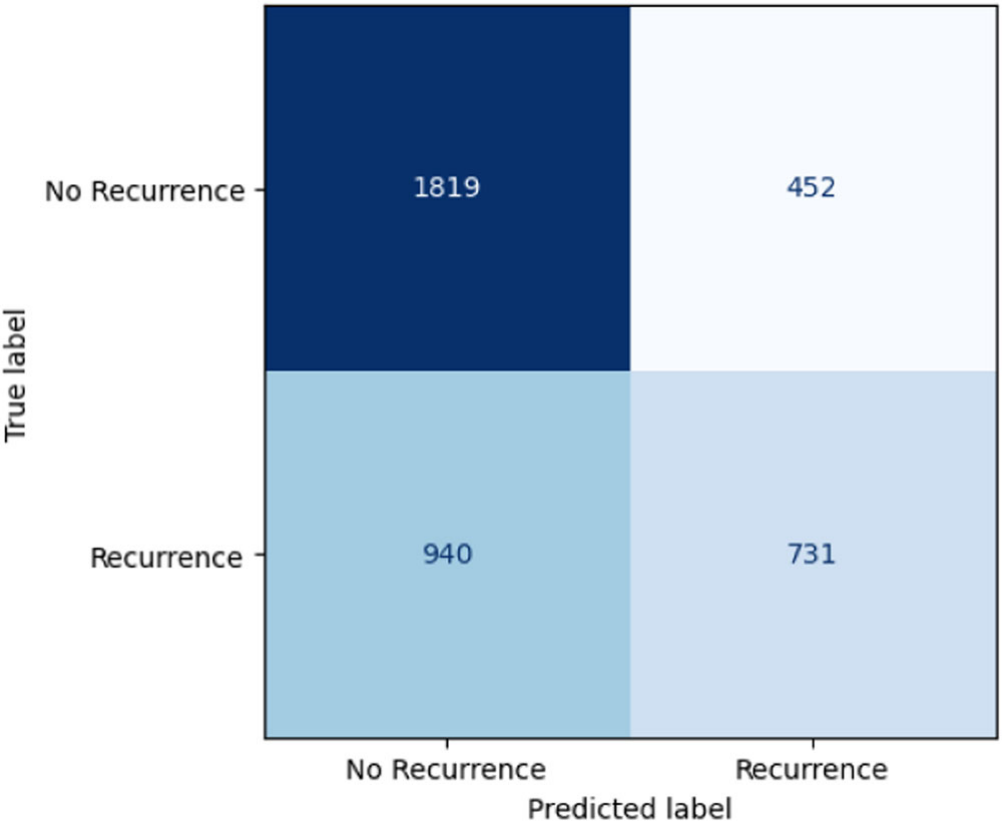
Confusion matrix. Showing the performance on the previously unseen test set using the default cut-off of 0.5. On the bottom right the cases are noted were recurrent contact was predicted and also occurred in reality (true positives). On the bottom left the cases of recurrent contact that were predicted wrongly are shown (false negatives). On the top left the correctly predicted no-recurrent chatters in the testset are counted (true negatives). Finally, on the top right the wrongly predicted recurrent chatters can be found (false positives).

We used the trained classifier for a one-time prediction on the test set consisting of the 3942 newest first consultations. Here we achieved an AUROC score of 0.68, which was significantly above randomness (*p* < 0.01). With the default threshold of 0.5 for the class probability, this led to a balanced accuracy of 0.62 and an accuracy of 0.65 (vs. baseline of 0.58). Therefore, 2550 of the 3942 new chatters would have been classified correctly. The precision here was 0.62, the recall 0.44. Hence, offering additional treatment based on this cut-off would have reached slightly below half of those that otherwise would have recontacted, and 62% of those with offered support would otherwise have been in recurrent contact. Sensitivity and specificity at this threshold were 0.62 and 0.66, respectively. (see Confusion Matrix in Fig. 2).

### Explainability

We used SHAP values as an explainability framework^38^, using the tree explainer^39^. In NLP, a challenge towards explainability is the large number of predictors, such as different word stems in our case^40^. Therefore, besides investigating SHAP values for single words we also applied a clustering approach, as specified in the Methods section.

**Fig. 3:**
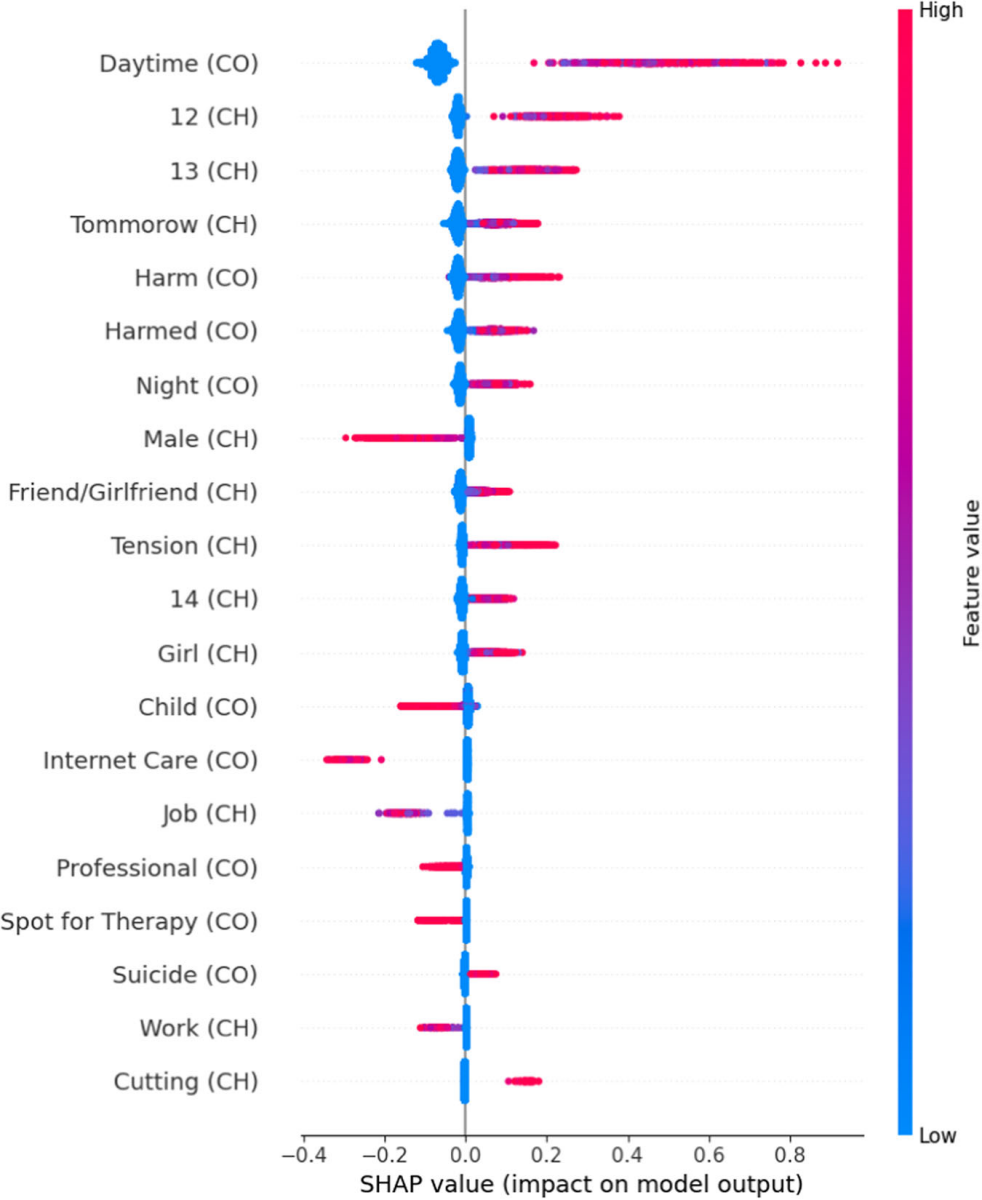
SHAP values for the discussed word stems, with CH/CO indicating whether the word was used by the chatter or the counselor. High feature values imply a positive impact on the prediction, negative feature values the opposite. Ordered by overall importance, but not including every word.

Overall, 1766 distinct word stems of the chatters and 1,186 of the counselor were included as predictors. Among the strongest predictors, according to their SHAP values, there were several ones indicating a young age of the chatter and being associated with higher chance of recontact. For example, “12” and “13” both had high SHAP values. Also, wordstems associated with “harm” (german: “verletzt”), particularly used by the counselor, predicted recontact. This indicates that chatters that struggled with self-harm behavior and thoughts have been more prone to message the service again. Wordstems related to time, such as “Daytime” (german: “tagsub”), “night” (“nacht”) were also among the words with high SHAP values, possibly indicating that the conversation might have ended due to the time of the day, e.g. as chatters hat to go to bed, and chatters recontacted in the following days. The wordstems “dying” (“sterben”) by the chatter and “suicide” (“suizid”, “selbstmord”) by the counselor predicted recontact, showing a relation between suicidal thoughts and ideation and need for help. Finally, words indicating female sex were associated with higher chance of contact, while the word “male” (“männlich”) predicted no recontact, indicating a gender-specific influence on the outcome. Additionally, several work and job-related words like “job” (“job”) or “work” (“arbeite”) by the chatter indicated a lower chance of recontacting. Finally, the terms “professional” (“professionell”), “internet care” (“internetseelsorg”) or “advice” (“rat”) also were predictors for no recurrent contact. As all these words were used by the counselor this could indicate already a form of redirection to other care. They also were an example for predictive signal possibly just being present on one side of the conversation and therefore contributing to the increase in performance when using counselors and chatters word stems simultaneously. The SHAP values for the mentioned word stems can be found in Fig. 3.

However, while this single word-based perspective provides interesting insight, several wordstems remain difficult to interpret. We therefore investigated what word stems most frequently co-occurred with the stems identified by the SHAP approach above, allowing additional insight about the context those were used in. As a deep dive into the use of the word “suicide” by counselors, we discovered the frequent co-occurence of the wordstem “thoughts” by the chatter. This strengthens the interpretation that counselors’ use of disorder-related words frequently was a reframing or clarification on problems shared by the chatters. However, interestingly, suicide also was used commonly in combination with the words “parents”, “mother” or “friend” by the chatter. indicating the possibility that chatters were not suicidal themselves but affected by a case in their social environment. We also looked into co-occurrences of the wordstem “harm” in counselor messages. In the approximately 5000 conversations where this stem was used by the counselor, we found the stem in the chatter messages for nearly 2/3 of those, substantiating the interpretation that counselors and chatters word mirror each other. This also fits the counseling strategy teached by the organization to new volunteers, encouraging validation and proactive listening. Finally, looking into work and job related words, we identified stems like “situation”, “flat”, “time” or “stress” as frequently co-occurring, providing some evidence for those words being related to everyday stress and struggles. Additional co-occurrences can be found in the supplementary table 1.

Finally, the overall number of predictors was high and even the 50 most influential wordstems contributed less than half of the total predictive performance, according to their SHAP values. Therefore, we clustered the included wordstems, using a pre-trained Word2Vec algorithm to group them by similarity. 1350 of the stems were present in the vocabulary of the used algorithm, for which we retrieved 20 clusters based on the silhouette score, including on average 67 wordstems. The most important cluster included numerous adjectives with strong emotional content such as “alone” (“allein”), “weak” (“schwach”), “wrong” (“falsch”) or “best” (“beste”). The second most relevant cluster included all numbers, strengthening the interpretation about the relevance of age for recontacting the service. Finally, the third cluster mainly consisted of nouns related to the life situation such as “work” (“arbeit”), university (“studium”), “city” (“stadt”) or “house” (“haus”). Notably, in line with the single stem perspective, also among the stems the predictive signal was widely spread with even the least relevant cluster having a notable contribution to the predictions. All clusters and their added up SHAP-values can be found in the supplementary Table 2.

## Discussion

The promise of NLP in (digital) mental healthcare to improve clinical decision-making and personalize treatment has to be matched with application-specific evaluations and feasibility studies. Therefore, this paper investigated a relevant use-case, the prediction of recurrent chat contact in a counseling hotline for the youth, as a relevant information point within a stepped care framework. Using anonymized chatdata with randomized word order in combination with a XGBoost classifier allowed us to reach a significant predictive performance on the unseen testset. Also, this approach outperformed a day-of-time and conversation-length-based baseline. SHAP values revealed age, gender, self-harm and suicidal thoughts as most important predictors.

The chosen approach outperformed numerous studies that also applied NLP for the prediction of outcomes in digital interventions. For example, Zantvoort and colleagues reported AUC scores of up to 0.64 using NLP techniques similar to the one in this paper, but also transformer-based approaches^41^. Another study reported a sensitivity of up to 0.59 and specificity of up to 0.60 for the NLP-only prediction of psychiatric outcomes at follow-ups^42^. Finally, a study reached an AUC of 0.57 for the prediction of binge eating for new users of an online intervention^43^. Also the comparison with meta-analytic evidence strengthens the feasibility of the results. The average accuracy of methodically adequate studies for predicting depression treatment outcome were reported to be 0.63 in a meta-analysis^44^ and therefore on a similar level to this study. For the prediction of the outcomes of cognitive behavioral therapy using machine learning, a pooled accuracy of 0.74 was reported^45^, which was slightly above the level of this study. However, notably, those reviews did not focus on text-only approaches and used a diverse set of predictors. The limited evidence from the same setting, chat-based counseling services, mostly reported very high predictive performances, such as an AUC of 0.9 for distress classification^27^ and recall scores of close to 0.9 for detecting suicidal crisis^28^. While impressive in their domain, we argue that those are not good baselines of comparison for the prediction of outcomes like recurrent chat contact. The key difference between those two studies and the approach in this paper is that they focus on chatters’ state during the moment of counseling. In contrast, outcome prediction for precision care focuses on predictive performance ahead of relevant outcomes, therefore with a time gap between train data and labels. In conclusion, we argue that in comparison with benchmarks from the literature this use case is promising as being competitive against NLP-based outcome prediction studies as well as meta-analytic evidence. However, more work is needed for similar prediction tasks in chat-counseling setups.

Another central benchmark for the feasibility of AI use cases is the clinical utility. Several factors for that, such as the ability to outperform the intuition of clinicians or the ease of data collection of the needed data, have been stated^45^. Interestingly, comparisons of human benchmarks for algorithms have been done very rarely in the field of clinical psychology (for example, for alcohol disorders^46^) and appear needed for a better understanding of the contribution of AI to mental healthcare. Future research comparing the performance in predicting recurrent chatting between the algorithm and the counselors of the service seems therefore highly relevant. This would possibly also enable a better understanding of predictive markers used exclusively either by the algorithm or the counselors. However, we believe that a reliable evaluation of the utility is actually an empirical question that can not be derived from either the predictive performance alone (even in comparison with clinicians) or theoretical considerations. Specifically, trials are needed that investigate whether the use of NLP/AI based predictions actually benefits outcomes. Trials comparing AI-informed treatment arms with standard care regularly report null results^47^. This highlights that explicit and use-case-specific testing of the clinical benefits of AI is needed.

Related to the clinical utility of the chosen approach, an open question is what intervention is actually most promising to be based on the predicted outcome. While we introduced several ideas across the paper, such as to proactive redirection to routine mental healthcare structures, more work appears needed toward actual implementation. A more granular insight into the reasons for recontacting could be a starting point to understand what form of prediction-based intervention would be most helpful. For example, chatters with additional need for information would possibly require a different follow-up than those needing more emotional support^48^. Also, there might be differences in need depending on how soon chatters recontacted the service. Therefore qualitative analyses of the reasons for recurrent chatting would be highly beneficial.

We implemented, influenced by data privacy considerations, a non-transformer-based algorithmic approach using single-word stems as predictors. This approach had several benefits worthwhile mentioning. The chosen approach works with heavily anonymized and preprocessed data. As our results, namely the chosen hyperparameters of the word vectorizer, showed, even stronger preprocessing would be possible without influencing the performance. Also, the use of counselors’ messages alone did exhibit promising predictive performance, potentially allowing to not use chatters messages at all in particularly data-privacy sensitive settings. Adolescent mental health data is particularly sensitive. Therefore, we see this as a large benefit for reusability of this approach as the need of storing and processing personal identifiable information (PII) is reduced. Also, the comparable low computational costs of training and tuning the algorithm make extensive cross-validation principles possible, contributing to the generalizability and preventing overfitting. On the other hand, the chosen approach did not have the capability to extract predictive signals from more complex language units than wordstems. Therefore, the application of pre-trained and transformer-based language models like BERT seems promising for ethically and legally less restricted settings and datasets. However, notably the use of more complex algorithmic approaches did not always lead to significantly improved predictive performance in digital mental health settings^41^. Recent evidence also showed that a simple compressor method for text classification outperforms BERT models for certain use cases^49^, highlighting that there is no deterministic relationship between the sophistication of an algorithmic approach and its performance. Therefore we believe that there is promise in the evaluation of transformer-based approaches in future research, but want to highlight the importance of including simpler approaches as baselines, in order to empirically investigate whether the increased algorithmic complexity actually provides improved performance.

There were limitations to the approach of this paper. Firstly, the counselors were never explicitly instructed to limit the number of consultations per chatter. Therefore, there were occasions where they proactively invited help seekers to contact the service again for additional help. In these cases recurrent contact was likely more indicative of counselors behavior and less of the need for chatter. For example, there were occasions where children were sent to bed and asked to recontact in the next morning, to not disrupt their sleep cycle. However, due to relatively low share of nightly contacts as well as the general availability to provide nightly counseling, those cases were rare. Secondly, the need for help is arguably not the only influence on the chance of recontacting. For example, while words related to male sex made recontact less likely this should not be misread as a lower need of follow-up help for male chatters. Possibly there were gender-specific influences on the satisfaction with the service that influenced recontact, independent of the need of additional help. Also, the inability to reconnect chatters with the same counselor is proactively communicated to the help seekers, which may have discouraged some helpseekers with unmet needs from returning.

Thirdly, while it is likely that most chatters with unmet help needs will contact the service again, there may be chatters whose dissatisfaction with the first contact stops them from recurrent chatting. This means there may be some further unmet need that we cannot address with our outcome. However, past evaluation efforts showed that around 2⁄3 of the chatters reported high satisfaction and nearly 90% a high likelihood to recommend the service^32^, suggesting that this may have been the case only for very few chatters.

Fourthly, while our outcome seems promising in capturing unmet additional need for help, it is weaker associated with absolute symptom severity. For example, when a chatter with severe problems already accessed help from e.g. a psychiatrist after a consultation, this person would be less likely to recontact the service. Including an additional symptom severity outcome would open up further treatment and redirection options for the chatservice and additional avenues for research. Fifthly, the applied anonymization principles also influenced our ability to interpret the model. Several words with high SHAP values were hard to interpret on their own, but due to data being shuffled it was not possible to look up the exact contexts the words were used in. Also, we were not able to investigate word bi-grams or tri-grams. Sixthly, a highly relevant challenge in NLP also affecting this study is biases that are embedded in pretrained models like the word2vec model used for the clustering approach^50,51^. Finally, we applied a time-based train test split as a validation principle. While providing improved external validity, there are also downsides of this technique. For example, seasonal influences would bias the performance in such an approach particularly strongly.

In conclusion, the text-only prediction of recurrent chat contact has been shown to be a promising NLP use case for chat-based counseling services. We hope that this work serves as a starting point for future work, such as the combination with non-text features, the testing of transformer-based algorithms, comparisons with a human baseline and the adaptation and evaluation of interventions on top of the prediction.

## Methods

This study was preregistered on OSF as a secondary data analysis (https://osf.io/xa4pn). Due to data privacy concerns coming up during the conceptualization of this paper there was a major update to this pre-registration. Against the initial proposal the available data had to be significantly more preprocessed in order to protect the rights of the chatters. As a consequence, we decided against evaluating transformer-based and Word2Vec algorithms, as the processing would have significantly biased the performance of those approaches. Specifically, the anonymization procedure made information on the order of words unavailable (see Preprocessing), which is influencing the performance of the vectorization and transformer-based approach but not of the word-count method. Additionally, to ensure the nature of this study as a secondary data analysis, we decided to exclude all data from before October 2021, which was the starting point for the data collection period approved in a past ethical approval (Ethical Committee University Leipzig, 372/21-ek). All chatters in the sample agreed with the use of their data for research purposes and therefore provided written informed consent before the start of their consultation. While data sharing is not possible for privacy reasons, the used code can be found on Github (https://github.com/silvanhornstein/RecurrentPrediction).

### Setting and intervention

Routine-care data from the German nonprofit organization “krisenchat” (https://krisenchat.de/) was used. krisenchat is a 24/7 available chat-counseling service that is provided free of charge for young people up to the age of 24^13^. Counselors ask for the first name, gender and age of the chatters, but providing those is not mandatory for receiving support. Counseling is provided mostly by extensively trained volunteers that have a background in psychology, psychotherapy, or social work. Counselors have a rich library of resources and exercises available to use with the chatters. One the one hand, links to youtube videos (e.g. https://www.youtube.com/watch?v=beO8PR7kIdo) or blog posts (e.g. https://krisenchat.de/oase/article/entscheidungen-treffen-hilfe) with psychoeducative content or exercises are shared. On the other hand, exercises like guided meditations are directly initiated and lead through the chat. Besides the provision of content, the counselors are trained to provide emotional and practical support as needed. Also, they provide information about the mental healthcare system as well as contact points for further help. Additionally, there is a crisis intervention team available as well as special teams with expertise for topics like child welfare endangerment, available on request by the counselor. This team also intervenes in cases of acute suicidal behaviour, applying a de-escalating strategy but if needed also contacting the local police. Due to the character of the intervention as first-point of contact, providing long-time support is currently not seen as responsibility of the service, as observable e.g. in the inability to rematch chatters with the same counselors when recontacting the service.

### Sample

We included all chatters that contacted krisenchat for the first time between October 2021 and December 2022. We decided to exclude those chatters that did not receive a consultation, which we defined as the reception of at least 3 messages from a counselor as well as an exchange of at least 10 messages. We based this criterion on qualitative insights into single chats as well as the intern logic used by the organization. After excluding 8419 individuals (31%), this led to a final sample of 18,871 unique chatters. Common reasons for contacts without a consultation were lack of capacities or chatters that did not respond to the counselor’s messages. We applied a time based train-test split and used the 14,931 chatters that received a consultation before September 2022 as train set and saved the newest 3,942 chatters (21%, consultation between September 2022 and December 2022) for a one-time final evaluation of the algorithm’s performance. As reported elsewhere^14^, those contacting krisenchat are mostly female and aged on average around 17, with psychiatric symptoms as the most likely reason for contacting the service. On average, the first consultation of chatters consisted of 25.4 messages of the chatter (SD = 20.8) and 17.7 by the counselor (SD = 12.1). Typically, 456.4 words (SD = 283.9) were exchanged with the longest conversation consisting of 3340 words. Comparison of these metrics between train - and test set revealed that consultations were longer in the test set, with a mean word difference of 34 words. More periods of high demand during the train period were named by counselors as a potential underlying cause of that difference.

### Outcome Variable

We decided for the recurrence of chat contact within half a year after the first consultation as an outcome. A consultation was defined as being completed if there was no message for 6 hours. Therefore, when a chatter message the service again in the morning after less than 6 hours since the last message, this did not count as recontact of the service. The decision for the exact timeframe of the outcome (188 days) was based firstly on the observations that a huge majority of recurrent contacts appeared within this timeframe. Finally, practical implications such as the legally allowed time to store chat conversations discouraged us further from including very late recurrent contacts into our outcome. Out of the 18,871 chatters in this sample, 8141 (43.1%) recontacted the service within 188 days. The chance of recontact did not differ significantly between the train and test set (43.3% vs. 42.3%; *p* = 0.29). More than half of the recurrent contacts occurred within the first week after their first consultation. Following the input of a reviewer, we also performed a sensitivity analysis to investigate whether the algorithm’s performance depended on the exact outcome criterion. Just minor variation (SD of the ROC AUC score of < 01) was reported across cut-offs spanning from 150 to 210 days.

### Data preprocessing and anonymization

Both the organization as well as the researchers involved in this project agreed on the rights of the highly vulnerable sample of this study being of top priority. Therefore, comprehensive preprocessing and anonymization techniques were applied, even if this led to a re-design of the originally proposed analysis (see preregistration). The proposed techniques were iterated and discussed with the data privacy lawyer of the organization to ensure alignment with the current law in Germany. As a first process for ensuring anonymized data, counselors were able to mark-personal identifiable information (PII) exchanged in a chat of them for deletion by the tech team. Secondly, all names were replaced with the token [NAME] and all city names with the token [CITY]. Thirdly, conversations were stemmed to reduce the re-identifiability of the users, e.g. by eliminating unique spelling patterns. Fourthly, the stemmed words were shuffled into random order within chatters and counselors, reducing the reidentifiability of narratives. Finally, every word that was present in less than 5 chats was deleted, so no word resulted to be an unique identifier of a certain consultation. In conclusion, krisenchat provided the data in the form of word stems in randomized orders, with unique identifiers eliminated, as a procedure to protect the rights of the young adults and children that searched for help at the service. The analysis of the data from the chat counseling service was approved by the ethics committee of the University of Leipzig (372/21-ek). We also presented this project to the ethics committee of the Humboldt-Universität zu Berlin, which confirmed that the analysis of fully anonymized secondary data does not require additional approval.

### Training

Due to the privacy-sensitive preprocessing described above, our approach focused on the Frequency-Inverse Document Frequency (TF-IDF) method to generate features from text. Each chat is represented as a *d*-dimensional vector where *d* is the number of unique wordstems appearing in all training chats. Each coordinate aims to represent the importance of a specific word for the given chat. A naive technique would be to concatenate all messages in a chat and retrieve the TF-IDF vector. However, any information on who used this word would be lost. Motivated by the assumption that certain words used by the counselor could be more important than if they are used by the chatter and vice versa, we decide to consider two TF-IDF vectors–one representing texts written by the chatter and another one for the messages of the counselor. We then used the concatenated TF-IDF vectors as inputs for a XGBoost classifier - a supervised machine learning algorithm that applies boosting and pruning^52^ which achieves state-of-the-art scores for diverse AI applications^53^. As a metadata-based baseline, we also trained an algorithm on the count of the word stems of the chatters and counselors, as well as the time of the day. For comparison, we applied a two-sided 5 × 2 test with corrected *p* value^54^ to evaluate the text-based approach against this baseline. Our training dataset consists of the 14,931 consultations that happened before September 2022. The hyperparameters of the vectorizer and classifier (see Table 2) are tuned simultaneously using a Bayesian search strategy over 250 repetitions using a repeated 5-fold cross-validation principle and optimizing the ROC AUC score. To implement our approach, we use the Python packages scikit-optimize^55^ respectively xgb^52^.

### Final evaluation

The test set consisting of the 3942 chatters with their first consultation between September and December 2022 was used for a final performance evaluation. The use of a time-based one-time prediction was done to improve the ecological validity of the approach, as the training of existing data for the prediction of new incoming data resamples the process of implementing AI solutions in real-world settings. Final performance was calculated by training the algorithms with the best-performing hyperparameters during training on the whole train data and predicting the outcomes for the test data once. We performed an additional evaluation of hypothesis 1 using a permutation test^56^ Therefore, after testing that the text-based algorithm can outperform a simple baseline over cross-validation, we here test the significance of the final performance against randomness.

### Explainability

SHAP values were used as an explainability framework. This approach building on game-theory uses the average contribution for every word stem used as a feature, for every prediction that was made. This estimation is then used as a base for evaluating the feature relevance. As the randomized order of our text data made the investigation of bi-grams and trigrams impossible, we used co-occurence of word stems as another explainability approach. Specifically, we investigated co-occuring word stems for the most relevant predictors in our approach.

Finally, we applied a clustering approach as an approach being able to handle large numbers of predictors. Specifically, a Word2Vec algorithm pre-trained on German wikipedia (https://github.com/devmount/GermanWordEmbeddings) corpora was used to retrieve values on 300 vector representations for every word stem. Those were then clustered, using a k-mean clustering approach. The decision for the number of clusters applied was based on a combination of usability (as to little clusters would not have been helpful) and the plot of the silhouette score.

### Reporting summary

Further information on research design is available in the Nature Research Reporting Summary linked to this article.

### Supplementary information

Supplementary Information

Reporting Summary

## Data Availability

Due to legal restrictions and in order to protect the sample of this study, the data can not be shared publicly. However, qualified researchers can apply for data access by contacting silvan.hornstein@krisenchat.de.

## Code availability

The code of this papers analysis can be accessed through github without restrictions: https://github.com/silvanhornstein/RecurrentPrediction.
