# Supplementary material for: Predicting recurrent chat contact in a psychological intervention for the youth using natural language processing
Source: NPJ Digit Med. 2024 May 18;7:132. doi: 10.1038/s41746-024-01121-9 (PMC11102489; doi:10.1038/s41746-024-01121-9)
Supplement: Supplementary file 1 — Supplementary Information [file 41746_2024_1121_MOESM1_ESM.pdf]

Supplementary Table 1
Clusters of Wordstems.

Exemplary Word Stems (German)	Exemplary Word Stems (English)	Overall Stems	Sum of SHAP Values
allein, belastet, besorgt, falsch, gezwungen, privat, schaden, verfolgt, verletzt, zeitlich	alone, burdened, worried, wrong, forced, private, damage, tracked, hurt, timely	194	610.3
10, 11, 12, 13, 14, 15, 16, 17, 18, 20	10, 11, 12, 13, 14, 15, 16, 17, 18, 20	19	541.3
aktion, arbeit, auto, besuch, bilder, freizeit, gruppe, haus, platz, uni	action, work, car, visit, pictures, leisure time, group, house, place, university	83	493.4
ablenken, akzeptieren, aufgeben, beruhigen, fahren, informieren, kontrollieren, sitzen, trennen, vermitteln	distract, accept, give up, calm down, drive, inform, control, sit, separate, convey	144	454.3
2020, abgelenkt, beide, daheim, gefunden, gespielt, kurz, unterwegs, wenige, zuletzt	2020, distracted, both, at home, found, played, short, on the way, little, last	211	353.3
achtsam, alt, deprimiert, ehrlich, herz, lachen, leid, ritzen, schwul, verliebt	mindful, old, depressed, honest, heart, laughing, suffer, scratch, gay, in love	89	265.4
abgemacht, angelogen, behauptet, entschuldigen, fragen, gegoogelt, mitbekommen, vergessen, wissen, woher	agreed, lied to, claimed, apologized, asked, googled, noticed, forgot, knew where from	96	258.8
arbeite, bereue, bleibe, empfinde, kenne, laufe, nehme, probiere, rede, studiere	work, repent, stay, feel, know, run, take, try, talk, study	60	162.2
angst, bleibst, denkst, doll, heulen, krieg. sagst, schlaf, spiel, verlierst	fear, stay, think, doll, cry, war, say, sleep, play, lose	64	161.5
achtet, anbietet, beginnt, bekommt, dreht, ergibt, funktioniert, gilt, kostet, ruft	pays attention, offers, begins, gets, turns, results, works, applies, costs, calls	67	141.6
as, back, film, job, moment, name, party, person, sex, situation	as, back, film, job, moment, name, party, person, sex, situation	26	103.4

allzu, angenehm, dunkel, eng, fit, freundlich, gelassen, kraftvoll, physisch, rational	all too, pleasant, dark, tight, fit, friendly, calm, powerful, physical, rational	87	97.4
besser, bessere, beste, gleiche, klasse, richtig, schlechte, schnelle, schwierige, verdient	too, pleasant, dark, tight, fit, friendly, calm, powerful, physical, rationalbetter, better, best, same, class, right, bad, fast, difficult, deserved	30	74.6
ah, bild, buch, chat, corona, hort, kinder, mama, mensch, tanz	ah, picture, book, chat, corona, after-school care, children, mom, human, dance	43	44.5
bedroht, behandelt, betrogen, diagnostiziert, geboren, schwanger, seelisch, verschrieb, verstorben, verschied	threatened, treated, cheated on, diagnosed, born, pregnant, mentally, prescribed, deceased, different	28	21.8
abwarten, bedanken, endlich, hinbekommen, hoffe, klappen, losgeht, riesig, stolz, weitermacht	wait, thank, finally, get it done, hope, it works, let's go, huge, proud, carry on	20	17.0
app, art, chance, box, form, hang, internet, option, partner, web	app, art, chance, box, form, tendency, internet, option, partner, web	36	16.9
abends, angeschrien, ausgezogen, einschlafen, essen, geheult, geschlafen, grundlos, trinken, vergewaltigt	in the evening, yelled at, undressed, fell asleep, ate, cried, slept for no reason, drank, raped	15	10.1
bereitet, sorge, sorgen, sorgt	prepares, cares, worries, worries	4	6.9

Supplementary Table 2
Overview of co-occurring word stems

	Co-Occurrences Chatter	Co-Occurrences Counselor
Daytime (CO)	time, fear, hello, feel, years, friend, school	situation, speak, year, night, evening, person, nature
12 (CH)	again, always, mother, time, parents, fear, school	situation, age, strong, help, important, someone, speak
13 (CH)	again, thinking, mother, time, parents, fear, school	situation, age, year, help, important, friend, great
Tomorrow (CH)	make, mine, time, mother, thanks, school, today	fear, situation, help, speak, relevant, therapy, head
Harm (CO)	fear, harm, feeling, school, thoughts, day, alone	right now, maybe, situation, help, strong, year, relevant
Night (CO)	again, everything, mine, fear, thanks, parents, sadly	fear, situation, year, age, friend, speak, believe
Male (CH)	time, girlfriend, speak, thoughts, mother, life, parents	maybe, situation, girlfriend, speak, gender, person, therapy
Friend/Girlfriend (CH)	always, again, speak, school, best, together, alone	situation, fear, relevant, speak, relationship, together, burdened
Tension (CH)	again, always, mine, fear, relaxation, thoughts, believe	tension, help, fear, situation, okay, strong, little
14 (CH)	always, again, everything, parents, mother, time, fear	situation, fear, speak, friend, family, therapy, family
Girl (CH)	always, know, again, do, speak, believe, problem	speak, help, someone, thing, regularly, young, wrote
Child (CO)	mine, mother, nothing, parents, age, feel, father	situation, fear, help, believe, family, nature, write
Internet Care (CO)	suicide, solution, always, again, mine, knowing, help	suicide, child, problem, young, different, situation, wait
Job (CH)	always, again, time, years, day, life, problems	situation, work, strong, speak, stress, therapy, positive
Professional (CO)	always, everything, time, fear, help, years, life	relevant, speak, therapy, burdened, step, topic, try
Spot for Therapy (CO)	therapy, mother, parents, help, feeling, day, problems	therapy, fear, year, help, therapist, together, step
Suicide (CO)	thoughts, parents, time, mother, life, suicide, school	situation, strong, help, clinic, therapist, family, night
Work (CH)	fear, years, school, dasy, problems, weeks, together	situation, speak, relevant, message, stress, positive, normal

Cutting (CH)	always, parents, feel, thoughts, friend, alone, write	strong, fear, situation, help, someone, speak, person
