# Supplementary material for: Predicting recurrent chat contact in a psychological intervention for the youth using natural language processing
Source: NPJ Digit Med. 2024 May 18;7:132. doi: 10.1038/s41746-024-01121-9 (PMC11102489; doi:10.1038/s41746-024-01121-9)
Supplement: Supplementary file 2 — Reporting Summary [file 41746_2024_1121_MOESM2_ESM.pdf]

nature portfolio

Corresponding author(s): DAPR - NPJDIGITALMED-08242

Last updated by author(s): Apr 3, 2024

Reporting Summary

Nature Portfolio wishes to improve the reproducibility of the work that we publish. This form provides structure for consistency and transparency in reporting. For further information on Nature Portfolio policies, see our Editorial Policies and the Editorial Policy Checklist.

Statistics

For all statistical analyses, confirm that the following items are present in the figure legend, table legend, main text, or Methods section.

n/a	Confirmed	
☐	☒	The exact sample size (*n*) for each experimental group/condition, given as a discrete number and unit of measurement
☒	☐	A statement on whether measurements were taken from distinct samples or whether the same sample was measured repeatedly
☐	☒	The statistical test(s) used AND whether they are one- or two-sided<br>*Only common tests should be described solely by name; describe more complex techniques in the Methods section.*
☒	☐	A description of all covariates tested
☐	☒	A description of any assumptions or corrections, such as tests of normality and adjustment for multiple comparisons
☐	☒	A full description of the statistical parameters including central tendency (e.g. means) or other basic estimates (e.g. regression coefficient) AND variation (e.g. standard deviation) or associated estimates of uncertainty (e.g. confidence intervals)
☐	☒	For null hypothesis testing, the test statistic (e.g. *F*, *t*, *r*) with confidence intervals, effect sizes, degrees of freedom and *P* value noted<br>*Give P values as exact values whenever suitable.*
☒	☐	For Bayesian analysis, information on the choice of priors and Markov chain Monte Carlo settings
☒	☐	For hierarchical and complex designs, identification of the appropriate level for tests and full reporting of outcomes
☒	☐	Estimates of effect sizes (e.g. Cohen's *d*, Pearson's *r*), indicating how they were calculated

Our web collection on statistics for biologists contains articles on many of the points above.

Software and code

Policy information about availability of computer code

Data collection	Chatters use Whatsapp to receive a consultation. Counselors use a self-build software tool. All conversations are stored securely in the organizations backend.
Data analysis	xgboost 1.7.4, shap 0.41.0, gensim 4.3.1, scikit-optimize 0.9.0, scikit-learn 1.2.1, (all python packages), python version 3.9.13

For manuscripts utilizing custom algorithms or software that are central to the research but not yet described in published literature, software must be made available to editors and reviewers. We strongly encourage code deposition in a community repository (e.g. GitHub). See the Nature Portfolio guidelines for submitting code & software for further information.

Data

Policy information about availability of data

All manuscripts must include a data availability statement. This statement should provide the following information, where applicable:

- Accession codes, unique identifiers, or web links for publicly available datasets
- A description of any restrictions on data availability
- For clinical datasets or third party data, please ensure that the statement adheres to our policy

Due to legal restrictions and in order to protect the sample of this study, the data can not be shared publicly. However, qualified researchers can apply for data access by contacting silvan.hornstein@krisenchat.de

Research involving human participants, their data, or biological material

Policy information about studies with human participants or human data. See also policy information about sex, gender (identity/presentation), and sexual orientation and race, ethnicity and racism.

Reporting on sex and gender	For this project we worked with naturalistic chat conversations. We are referring to "gender" when speaking about cases where e.g. "female" as term was used. We do this, assuming that this mostly is a self-reference by the chatters, for which gender seems the more appropriate term.
Reporting on race, ethnicity, or other socially relevant groupings	Working with the verbal material used by those contacting the service investigated in our study, we did not constructed any explicit variables on race, ethnicity or other socially relevant groupings. Among the predictors reported there is none that we would classify as being related to this categories.
Population characteristics	As a naturalistic study, there was no explicit sample selection. In contrast, every chatter that contacted the service was included when being consulted by the service. Those contacting are on average 17, mostly female, and mostly contacting due to psychiatric symptoms (see Eckert et al., 2022 for more details).
Recruitment	There was no recruitement, as a naturalistic sample from a chat-consultation service was used.
Ethics oversight	University of Leipzig (372/21-ek), Humboldt Universität zu Berlin

Note that full information on the approval of the study protocol must also be provided in the manuscript.

Field-specific reporting

Please select the one below that is the best fit for your research. If you are not sure, read the appropriate sections before making your selection.

☐ Life sciences ☒ Behavioural & social sciences ☐ Ecological, evolutionary & environmental sciences

For a reference copy of the document with all sections, see nature.com/documents/nr-reporting-summary-flat.pdf

Behavioural & social sciences study design

All studies must disclose on these points even when the disclosure is negative.

Study description	This Natural Language Processing Study uses around 800.000 messages from a low-threshold chat consultation service to predict who will predict the service again, using a XGBoost Classifier and Bayesian Hyperparametertuning.
Research sample	19,000 children and young adults that received a chat consultation from a 24/7 crisis service in Germany.
Sampling strategy	Naturalistic Sample, therefore everyone who received a consultation.
Data collection	This study solely works with naturalistic data, therefore the chatdata available at the service as well as whether they recontacted the service, and the time of their first consultation.
Timing	between October 2021 and December 2022
Data exclusions	We excluded 8,419 chatters that exchanged less than 10 messages/received less than 3 counselors message.
Non-participation	Some participants requested the deletion of their data from the organization and therefore did not entered our analysis.
Randomization	No Randomization Involved.

Reporting for specific materials, systems and methods

We require information from authors about some types of materials, experimental systems and methods used in many studies. Here, indicate whether each material, system or method listed is relevant to your study. If you are not sure if a list item applies to your research, read the appropriate section before selecting a response.

Materials & experimental systems

n/a	Involved in the study
☒	☐ Antibodies
☒	☐ Eukaryotic cell lines
☒	☐ Palaeontology and archaeology
☒	☐ Animals and other organisms
☐	☒ Clinical data
☒	☐ Dual use research of concern
☒	☐ Plants

Methods

n/a	Involved in the study
☒	☐ ChIP-seq
☒	☐ Flow cytometry
☒	☐ MRI-based neuroimaging

Clinical data

Policy information about clinical studies
All manuscripts should comply with the ICMJE guidelines for publication of clinical research and a completed CONSORT checklist must be included with all submissions.

Clinical trial registration	no Trial
Study protocol	https://osf.io/xa4pn
Data collection	Naturalistic Data from the Service between October 2021 and December 2022
Outcomes	As outcome, we investigated whether chatters recontacted the service within 6 months.

Plants

Seed stocks	*Report on the source of all seed stocks or other plant material used. If applicable, state the seed stock centre and catalogue number. If plant specimens were collected from the field, describe the collection location, date and sampling procedures.*
Novel plant genotypes	*Describe the methods by which all novel plant genotypes were produced. This includes those generated by transgenic approaches, gene editing, chemical/radiation-based mutagenesis and hybridization. For transgenic lines, describe the transformation method, the number of independent lines analyzed and the generation upon which experiments were performed. For gene-edited lines, describe the editor used, the endogenous sequence targeted for editing, the targeting guide RNA sequence (if applicable) and how the editor was applied.*
Authentication	*Describe any authentication procedures for each seed stock used or novel genotype generated. Describe any experiments used to assess the effect of a mutation and, where applicable, how potential secondary effects (e.g. second site T-DNA insertions, mosiacism, off-target gene editing) were examined.*
